# Fibromyalgia in the Era of Brain PET/CT Imaging

**DOI:** 10.3390/jcm14124166

**Published:** 2025-06-12

**Authors:** Elisabetta Abenavoli, Valentina Berti, Matilde Nerattini, Piercarlo Sarzi-Puttini, Georgios Filippou, Alessandro Lucia, Gilberto Pari, Stefano Pallanti, Fausto Salaffi, Marina Carotti, Silvia Sirotti, Francesco Porta

**Affiliations:** 1 Nuclear Medicine Division, Careggi University Hospital, 50134 Florence, Italy; 2 Nuclear Medicine Unit, Department of Experimental and Clinical Biomedical Sciences “Mario Serio”, University of Florence, 50134 Florence, Italy; valentina.berti@unifi.it (V.B.); matilde.nerattini@unifi.it (M.N.); 3 Department of Rheumatology, IRCCS Galeazzi Sant’Ambrogio Hospital, 20157 Milan, Italy; piercarlo.sarziputtini@gmail.com (P.S.-P.); gf.filippou@gmail.com (G.F.); silvia.sirotti@grupposandonato.it (S.S.); 4 Department of Biomedical and Clinical Sciences, University of Milan, 20122 Milan, Italy; 5 Department of Clinical Sciences and Community Health, University of Milan, 20122 Milan, Italy; alessandro.lucia@unimi.it; 6 Interdisciplinary Pain Medicine Unit, Santa Maria Maddalena Private Hospital, 45030 Occhiobello, Italy; g.pari@medicinadeldolore.org (G.P.); dr.fporta@gmail.com (F.P.); 7 Department of Psychiatry, Albert Einstein College of Medicine, 1300 Morris Park Ave, Bronx, NY 10461, USA; stefanopallanti@yahoo.it; 8 Institute of Neurosciences, 50121 Florence, Italy; 9 Department of Clinical and Molecular Sciences, “Carlo Urbani” Hospital, 60035 Jesi, Ancona, Italy; reumatologia@faustosalaffi.it; 10 Department of Radiology, Ospedali Riuniti, Università Politecnica Delle Marche, 60126 Ancona, Italy; marina.carotti@gmail.com

**Keywords:** fibromyalgia syndrome, brain, PET/CT, central nervous system

## Abstract

Fibromyalgia syndrome (FMS) is a complex, heterogeneous disorder characterized by chronic widespread pain, fatigue, and cognitive disturbances. The multifactorial nature of FMS, with the involvement of central and peripheral mechanisms, hampers diagnosis and effective treatment. In recent years, positron emission tomography (PET) imaging has emerged as a valuable tool for exploring the neurobiological underpinnings of FMS. Several studies have investigated alterations in glucose metabolism, neurotransmitter systems (including opioid, dopamine, and GABAergic pathways), and neuroinflammation using various PET tracers. These findings have revealed distinct brain metabolic and molecular patterns in FMS patients compared to healthy controls, particularly in pain-related regions such as the thalamus, insula, and anterior cingulate cortex (ACC). Moreover, preliminary data suggest that PET imaging may help identify FMS subgroups with different pathophysiological profiles, potentially allowing for tailored therapeutic approaches. This review summarizes the current evidence on PET applications in FMS and discusses the potential role of molecular imaging in improving patient stratification and predicting treatment response.

## 1. Introduction

### 1.1. Introduction

The clinical goal of the management of any disease is to ensure diagnostic procedures that can support an accurate diagnosis, allowing appropriate decision making and targeted therapy, thus improving patient outcomes.

FMS is a complex condition that is characterized by widespread pain, chronic fatigue, sleeping disorders, and cognitive deficits that are often known as “fibro-fog”, but may also involve a range of other symptoms such as headache, irritable bowel syndrome (IBS), temporomandibular joint disorders, and anxiety and depression [1,2,3]. One of its hallmarks is the presence of specific anatomical locations or “tender points” at which pain is caused by pressure [4]; however, this feature is no longer used as a diagnostic criterium. FMS mainly affects women and typically occurs during middle adulthood, although it may appear at any age [5].

Its pathophysiology is still unclear, but recent studies have shown that it may involve the interplay of various genetic, neurological and immunological factors [6,7,8,9]. The risk of developing the disease has been associated with various genetic markers, and it has been found that reduced serotonin levels and norepinephrine and dopamine dysregulation seem to be involved in FMS-related alterations in pain modulation and emotional factors [7,8,9]; furthermore, changes in the function of N-methyl-D-aspartate (NMDA) receptors and disequilibrium in the excitatory and inhibitory neurotransmitter systems are associated with central sensitization, a critical aspect of FMS [10]. Finally, emerging evidence suggests that immune system dysregulation, including elevated levels of pro-inflammatory cytokines, may also contribute to the pathophysiology of FMS [11,12,13].

Neuroimaging studies have identified key brain changes associated with pain processing. Structural MRI has revealed microstructural alterations in regions such as the thalamus and cortex [14], while functional MRI studies have demonstrated altered connectivity in areas like the insulae and somatosensory cortex [14], as well as increased insular activation in response to thumb pressure [15]. Metabolic dysregulation has also been reported, with reduced gray matter volume in the left insula [16] linked to neurotransmitter imbalances, including increased glutamate [17] and decreased GABA levels [18]. Additionally, dysfunction in the endogenous opioid system, such as altered connectivity between the periaqueductal gray matter and cortical pain-regulation areas [15], has been associated with reduced availability of μ-opioid receptors in the dorsolateral prefrontal and ACC [19].

This review examines the role of PET imaging in the clinical and research contexts of FMS, focusing on its ability to evaluate functional and structural alterations in the brain. PET imaging offers a sophisticated, non-invasive approach to assessing key pathological features of FMS, including altered metabolism, receptor density, neurotransmitter system dysregulation, and neural dysfunction.

With specific radio-labeled tracers targeting distinct neural pathways, PET enables detailed visualization of abnormalities across various cortical regions. This capability allows for the identification of disease-specific patterns, distinguishing FMS patients from healthy controls. Furthermore, PET imaging demonstrates potential as a biomarker for predicting clinical outcomes such as pain severity and quality of life, while also facilitating the identification of treatment responders and guiding individualized therapeutic strategies.

By integrating its findings with clinical data, PET imaging provides critical insights into FMS-related dysfunction and its underlying mechanisms. Moreover, its ability to detect treatment-induced changes highlights its potential to advance both the understanding and management of FMS.

### 1.2. PET Imaging

The goal of molecular imaging is to visualize specific molecules in living systems, helping to study molecular and cellular processes [15]. This includes:

1. Showing how a radiopharmaceutical spreads naturally in the body.
2. Understanding how a radiopharmaceutical interacts with its target.
3. Identifying key imaging patterns in diseases using visual analysis or measurements from specialized software.

In the last decade, PET imaging has become a powerful tool for detecting changes in the brain of patients with neurological diseases and evaluating new treatments [20,21,22,23].

**Figure 1:**
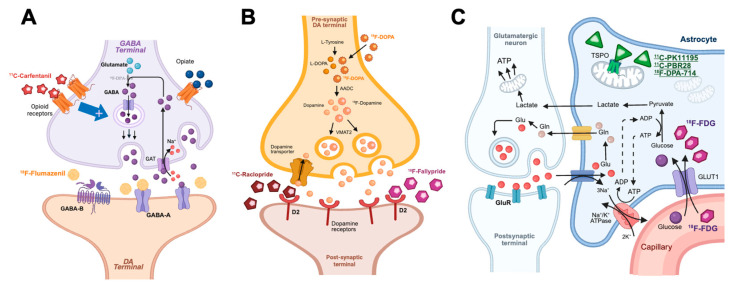
Principal PET radiopharmaceuticals and their targets investigated in FMS. (**A**) GABAergic synapses: [11C]Carfentanil targeting pre-synaptic opioid receptors, and [18F]Flumazenil targeting post-synaptic GABA_A_ receptors; (**B**) Dopaminergic synapses: [18F]DOPA targeting DA synthesis, and [11C]Raclopride or [18F]Fallypride targeting post-synaptic D2 receptors; (**C**) Glutamatergic synapses: [18F]FDG targeting glucose metabolism and synaptic function, and [11C]PK11195 targeting TSPO receptors. GABA: gamma-amino-butyric acid; GAT: GABA transporter; DA: dopamine/dopaminergic; D2: Dopamine receptor 2; VMAT: vesicular monoamine transporter; AADC: aromatic L-amino acid decarboxylase; [18F]DOPA: [18F]dihydroxyphenylalanine; [18F]FDG: [18F]fluorodeoxyglucose; GLUT: glucose transporter; Glu: glutamate; Gln: glutamine; ATP: adenosine triphosphate; ADP: adenosine diphosphate; GluR: glutamate receptor. Created using BioRender.com.

Statistical parametric mapping (SPM) is a method used to analyze brain scans and compare activity in different areas. It helps identify how radiotracers are distributed in the brain [24]. This technique has been used to study brain function in FMS patients (Figure 1) with the following tracers:

- [18F]FDG, a tracer of brain glucose metabolism and general (mainly glutamatergic) synaptic activity;
- Dopaminergic system tracers, including [11C]Raclopride or [18F]Fallypride to measure D2/D3-DA receptor availability, and [18F]DOPA to measure DA synthesis and metabolism;
- [18F]Flumazenil, a tracer of GABA_A_ receptors;
- [11C]Carfentanil, a ligand of μ-Opioid receptor (MOR);
- TSPO-targeting tracers, including the first-generation ligand [11C]PK11195, and second-generation radioligands [11C]PBR28 and [18F]DPA-714^.^

#### 1.2.1. PET Patterns in FMS: Diagnosis and Prognosis

PET may play a critical role in managing patients with suspected FMS by non-invasively and reliably distinguishing patients from controls (Table 1, Table 2 and Table 3).

**Figure 2:**
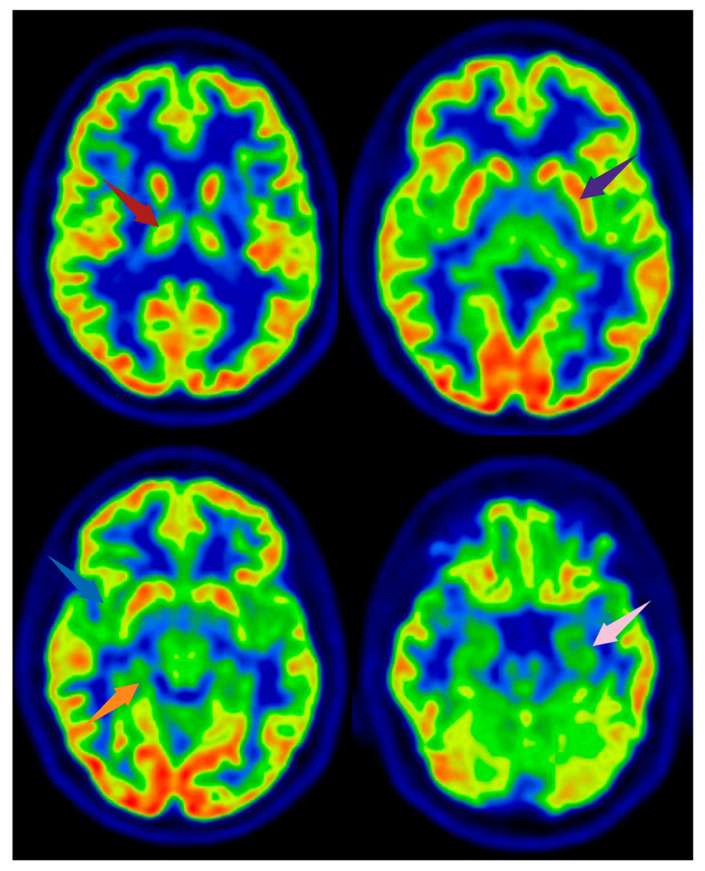
[18F]FDG PET images from a healthy subject. Arrows indicate brain regions commonly reported as altered in FMS, shown here for illustrative purposes. The subject depicted did not have FMS. Axial slices are shown. Red arrow: right thalamus; purple arrow: left lentiform nucleus; blue arrow: right insula; orange arrow: parahippocampal gyrus; pink arrow: medial temporal cortex. Image created using BioRender.com.

**Figure 3:**
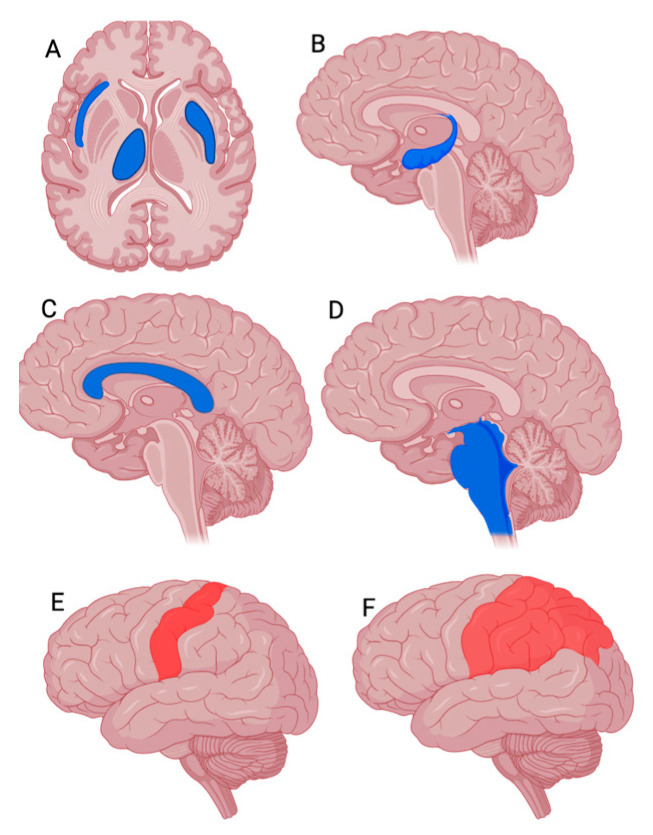
Representative PET findings in FMS patients compared to healthy controls. Created using BioRender.com. (**A**,**B**) [18F]FDG PET images (axial and sagittal slices): regions in blue indicate reduced glucose metabolism in the right thalamus, right insula, and left striatum (axial), and hippocampus (sagittal) in FMS patients. (**C**,**D**) [18F]DOPAPET images (sagittal slices): blue regions represent decreased presynaptic dopaminergic activity in the limbic system and brainstem. (**E**,**F**) TSPO PET images (e.g., [11C]PBR28 or [18F]DPA-714; sagittal slices): red regions indicate increased tracer uptake reflecting neuroinflammation in the primary somatosensory cortex and right parietal gray matter. Color scale interpretation: blue areas indicate reduced tracer uptake in FMS patients compared to healthy controls; red areas indicate increased uptake.

Several PET studies have investigated alterations in regional cerebral glucose metabolism in FMS patients using [18F]FDG. Most of the findings have been controversial, as they have failed to demonstrate any statistically significant difference in brain glucose metabolism between treatment-naïve FMS patients and healthy subjects [25,26] (Figure 2 and Figure 3).

The comparison of FMS patients with good and poor responses to medical pain reduction treatment revealed that different brain structures are involved in pain modulation in [18F]FDG PET studies [25,26]. It was found that poor responders show significantly greater metabolism in the left thalamus, bilateral lentiform nucleus, and right parahippocampal gyrus than good responders [27]. This is particularly interesting because an earlier study found that the same brain regions were involved in the analgesic effects of electroconvulsive therapy for FMS-related pain [28]. This effect has been linked to altered thalamic activity following transcranial direct current stimulation and complex changes in gray matter volume [29,30].

[18F]FDG PET scans have also revealed that the insular metabolic hypoactivity is associated with the development of hyperalgesia [31]. Peyron et al. have pointed out that the insular cortex as one of the regions most consistently activated by noxious stimuli related to intensity coding [32]. The insula is also a critical point in the interconnection of dopaminergic (DAergic) and gamma-aminobutyric acid (GABA) activity because GABAergic interneurons are involved in its DAergic modulation [33]. GABA greatly inhibits neuronal activity within the insula; thus, a reduction in insular GABAergic neurotransmission lowers the pain threshold and leads to the hyperalgesia observed in chronic pain conditions [27,34,35,36].

PET imaging can also reveal the imbalance between excitatory and inhibitory neurotransmission. Studies using [18F]Flumazenil, a tracer for GABA_A_ receptors, have shown that patients with FMS exhibit 11–31% higher receptor binding in brain regions associated with the attention and default mode networks compared to healthy controls, suggesting enhanced hypervigilance and impaired cognitive disengagement in FMS [37].

Presynaptic DA transporters (DATs), which remove DA from the synaptic cleft after phasic activation, also seem to play a role in pain modulation. Furthermore, one [18F]DOPA PET study has found that DA metabolism is significantly lower in FMS patients than in controls not only in pain cortex regions such as the brainstem, thalamus, and multiple areas of the limbic system, but also in the ventral tegmental area (VTA) [38] (Figure 2).

The modulation of the VTA is also influenced by GABAergic neurons through the activation of MOR [39,40].

PET studies of MOR tracers have demonstrated that MOR density is even lower in the amygdala, cingulate, and nucleus accumbens of FMS patients [41,42], thus suggesting that these structures are involved in the clinical presentation and possibly the continuation of painful symptoms.

Neuroinflammation has emerged as a pivotal pathological feature in FMS. Increasing evidence underscores the central role of glial activation, as indexed by TSPO, in the pathophysiology of FMS. A seminal PET study utilizing the radioligand [11C]PBR28 provided the first direct in vivo evidence of glial activation in FMS, demonstrating widespread cortical increases in TSPO expression, particularly in the frontal and parietal cortices, without corresponding elevation in astrocytic activity, as assessed by [11C] L-deprenyl-D2 PET. These findings were consistent with microglial activation as a central mechanism in FMS pathophysiology [43]. A subsequent investigation extended these findings, identifying specific elevations in TSPO binding in the primary somatosensory cortex, more pronounced in patients with radicular pain compared to axial back pain (Figure 3).

This elevation was positively correlated with thalamic connectivity and the severity of nociplastic pain (fibromyalgianess). Mediation analysis indicated that fibromyalgianess modulated the relationship between TSPO expression and somatosensory cortex–thalamus connectivity, underscoring the role of neuroinflammation in the centralization of pain [44]. Further supporting these observations, a third study employing the second-generation TSPO radioligand [18F]DPA-714 demonstrated increased TSPO binding in FMS patients relative to healthy controls, with significant increases observed in regions such as the postcentral gyrus, occipital, and temporal gray matter. In high-affinity binders, these elevations extended to areas like the precuneus, parietal gray matter, and supramarginal gyrus. Importantly, increases in TSPO binding in the right parietal gray matter were associated with diminished quality of life, greater pain severity, and cognitive dysfunction, reinforcing the role of neuroinflammation in the clinical manifestations of FMS [45]. Taken together, these studies provide compelling evidence for glial activation in FMS, refining our understanding of its neuroinflammatory signatures and emphasizing the potential of neuroimmune biomarkers for targeted pain management and therapeutic interventions [46].

#### 1.2.2. PET Scan Findings Correlate with the Results of Standardized Pain Testing Procedures and Quality of Life Questionnaires

FMS is characterized by an altered sensation of pain due to the amplification and/or decreased inhibition of pain stimuli at multiple levels in the nervous system, and its diagnosis is based on criteria that have evolved to reflect our greater understanding of the condition. The 1990 American College of Rheumatology (ACR) diagnostic criteria required the presence of chronic widespread pain and pain upon palpation in at least 11 of 18 specific bodily sites [47], whereas health professionals today primarily refer to two sets of criteria: the 2016 ACR criteria and the 2018 criteria of the American Academy of Pain Medicine (AAPT), which highlight different aspects of the disease.

Based on an update of the 2010 ACR criteria, the 2016 ACR criteria require the presence of generalized pain (i.e., pain in at least four of five bodily regions), a Widespread Pain Index (WPI) of >7 and a Symptom Severity Score (SS) of ≥5 or ≥9 if the WPI is 3–6; the pain must have persisted for at least three months and any other disorder that might explain it must be excluded [48,49]. The 2018 AAPT criteria expand the diagnostic framework by referring to a combination of multi-site pain (MSP) and moderate–severe mental and/or physical fatigue or sleep disturbances (difficulty in falling or staying asleep, and non-restorative sleep) [50].

The most widely used FMS assessment scales are the Fibromyalgia Impact Questionnaire (FIQ) or its revised version (FIQR), and the 36-item Short Form Health Survey (SF-36) [51]. The domains of the FIQ (a validated, disease-specific measure covering most disease-related symptoms that has been demonstrated to be sensitive to chance) are physical functioning, work status, depression, anxiety, sleep, pain, stiffness, fatigue, and well-being; the FIQR adds the domains of memory, tenderness, balance, and environmental sensitivity [52]. The SF-36 effectively discriminates patients with FMS from those with widespread pain alone, and healthy subjects from patients affected by either of the two painful conditions Its domains include physical functioning, role limitations due to physical health problems, bodily pain, general health perceptions, vitality (energy/fatigue), social functioning, role limitations due to emotional problems, and mental health [49].

**Figure 4:**
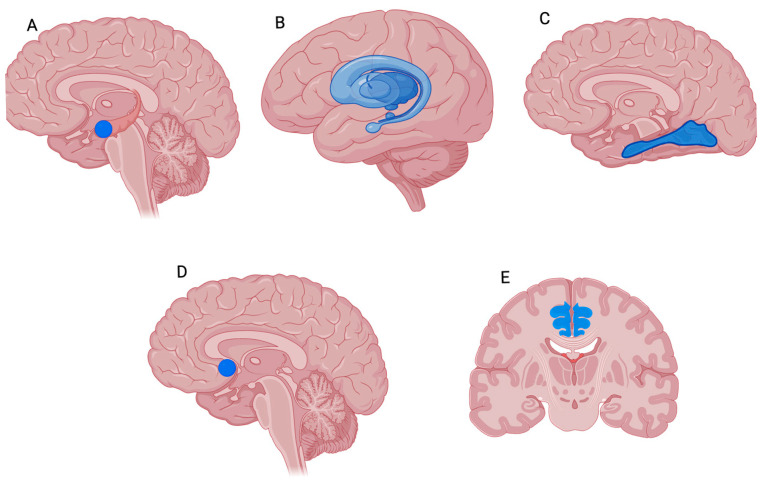
Representative PET findings in FMS patients compared to healthy controls. Created using BioRender.com. (**A**) [18F]FDG PET sagittal slice. Blue regions indicate reduced glucose metabolism in the amygdala of FMS patients compared to healthy controls. (**B**) [11C]Raclopride PET sagittal slice. Blue regions reflect decreased dopaminergic receptor availability in the basal ganglia. (**C**) [18F]Fallypride PET sagittal slice. Blue regions show reduced D2/D3 receptor binding in the fusiform gyrus. (**D**) [11C]Carfentanil PET sagittal slice. Blue regions represent lower MOR availability in the nucleus accumbens. (**E**) [11C]PK11195 PET coronal slice. Blue regions demonstrate reduced TSPO binding in the ACC of FMS patients. Color coding: Blue = reduced radiotracer uptake or receptor availability in FMS patients compared to healthy control.

In a comparison with healthy subjects, one [18F]FDG PET study found that FMS patients showed a distinct positive correlation between metabolism in the amygdala and tender point scores [28] (Figure 4). The amygdala is recognized as a structure involved in processing sensory stimuli and the cognitive-affective aspects of pain (memories and expectations) [48,50].

An [18F]Flumazenil PET study found that increased GABA_A_ receptor density in the insula positively correlated with functional status (FIQ score) and pain levels and negatively correlated with the results of a memory task. These findings suggest that altered receptor levels may be clinically relevant not only etiologically, but also as a contributory or therapeutic factor [51].

Wood et al. [53] used PET and [11C]Raclopride (a D2/D3 receptor ligand) to detect D2/D3 receptor availability in a study investigating endogenous DA release during painful stimulation. FMS patients and healthy controls received an injection of painful hypertonic or non-painful normal saline into the anterior tibialis muscle and as expected, the FMS patients found the hypertonic saline injection more painful than the controls. The FMS patients also showed a decrease in normal DA release in the basal ganglia during the painful stimulation instead of the increase experienced by healthy subjects, which suggests that DAergic modulation in the basal ganglia could become a possible therapeutic target (Figure 4).

Other studies have used [11C]Raclopride PET to test the correlation between striatal D2/D3 receptor availability and the response to painful stimuli in FMS patients [54,55].

As it is a chronic condition, the course of FMS is often complicated by major depressive disorder (MDD), which may affect more than half of all FMS patients, a much larger proportion than that observed in the general population [56]. MDD can increase the severity of FMS and often requires the consideration of additional treatments in a frequently futile attempt to optimize patient outcomes [57]. More specifically, MDD mediates the relationship between pain intensity and physical functioning in such a way that FMS patients with MDD experience higher pain levels and a poorer quality of sleep than those without MDD [58,59].

As MDD can influence the thermal pain threshold, one study has investigated its correlation with D2/D3 receptor availability in FMS patients with and without MDD, as well as in healthy controls, using [11C]Raclopride. The results demonstrated different levels of dopaminergic receptor availability across various subcortical regions, including the striatal nuclei, nucleus accumbens, and other associated areas, depending on whether individuals were healthy, had FMS without MDD or had FMS with MDD. No significant correlation among striatal D2/D3 receptor availability, thermal pain tolerance and discriminative stimuli capacity was found in any of the three groups. Finally, the patients with MDD could be distinguished from those without MDD by their lower uptake in the left ventral striatum, a subcortical structure associated with the emotional processing of pain [8,54].

Albrecht DS at al. [60] observed significant alterations in the dopaminergic system among individuals with FMS compared to healthy controls using [18F]Fallypride PET imaging. They reported that FMS patients displayed markedly lower DA D2/D3 receptor binding availability, with reductions of approximately 29.6% in regions such as the ACC and fusiform gyrus during a working memory task (Figure 4). This diminished receptor availability suggests impaired cortical dopaminergic signaling in FMS. Furthermore, subjective pain ratings in FMS patients were negatively associated with [18F]Fallypride binding potential in several regions, including the left orbitofrontal cortex and parahippocampal gyrus (Figure 4). These findings imply a direct relationship between reduced DA receptor activity and increased pain perception. Sensitivity and tolerance to experimentally induced pressure pain also negatively correlated with D2/D3 receptor availability in key areas such as the hippocampus, ACC, bilateral striatum, and inferior frontal gyrus in both FMS patients and healthy controls(Figure 4). However, distinct correlations were noted in healthy subjects, including additional involvement of the insula and thalamus, suggesting group-specific differences in pain modulation mechanisms.

Harris et al. [41] revealed a reduction in MOR binding potential (BP) images in FMS patients within structures typically observed in imaging studies of experimental pain involving healthy control participants. These pain-associated structures included the amygdala, the cingulate and the nucleus accumbens (Figure 4). They also found a negative correlation between MOR BP within the accumbens and clinical ratings in the affective dimension of pain.

Finally, new radio-labeled tracers whose target is translocator protein have become an important means of evaluating activated microglia and astrocytes [61]. Neuroinflammation has emerged as a critical pathophysiological mechanism in FMS, with recent studies elucidating both central and peripheral correlates of aberrant glial activation. Seo et al. utilized [11C]PK11195 PET imaging to demonstrate significant microglial activation in key brain regions—namely the thalamus, ACC, and prefrontal cortex—which correlated positively with affective pain (MPQ-A scores) and stress (SRI scores) [62] (Figure 4).

In a complementary investigation, researchers integrated [11C]PK11195 PET with magnetic resonance spectroscopy to examine associations between neuroinflammation and neurometabolic as well as peripheral biomarkers in FMS.

In this study, FMS patients exhibited significant negative correlations between neuroinflammatory indices (distribution volume ratios) and the creatine/total creatine ratios in both thalami, as well as between neuroinflammation and the glutamate/total creatine ratio in the right insula. Moreover, peripheral biomarkers such as blood creatinine and C-reactive protein were also examined, with creatinine levels showing significant negative correlations with neuroinflammation in the left thalamus and left insula. Moreover, neuroinflammatory measures in the left thalamus and left insula were inversely associated with peripheral creatinine levels, with additional peripheral parameters also showing significant correlations [63].

Collectively, these findings underscore that microglial activation in FMS is not only central to the process of pain centralization but also intricately linked to alterations in central neurometabolite levels and peripheral biochemical markers. This integrated approach reinforces the potential of neuroimmune biomarkers as targets for precision therapeutic interventions in FMS (Table 1, Table 2 and Table 3).

### 1.3. Post-Treatment PET Imaging

Despite the advances made in treatment options, failure is still a significant clinical issue. However, post-treatment PET imaging can offer precise information in the form of brain mapping (Table 1, Table 2 and Table 3).

A pilot study by Walitt et al. [64] used [18F]FDG PET to assess the correlation between symptom improvement after an individualized multidisciplinary therapeutic regimen and regional brain metabolism. Increases in metabolic activity within several limbic system structures were associated with concurrent improvements in symptoms as measured by FIQ. The absence of metabolic changes in somatosensory areas confirmed that the limbic system is a principal player in the pathogenesis of FMS. One limitation of the study is that the limbic system is a target of most neurological medications and the changes in metabolic activity may have been due to this factor. At the same time, it is important to point out that the neuroreceptor-targeted medications used in the different limbic regions also act on physical symptoms.

One randomized controlled trial involved FMS patients who underwent 14 sessions of high-frequency repetitive transcranial magnetic stimulation (rTMS) or sham stimulation of the left primary motor cortex over a period of ten weeks [65]. The participants also underwent [18F]FDG PET at baseline, in treatment week 2, and in post-treatment week 11, at the end of which changes in their quality of life were measured using both the FIQ and the SF-36. The results of the FIQ and the mental component of the SF-36 showed a greater improvement in the quality of life of the patients in the active rTMS arm, but the treatment had no significant impact on their pain or anxiety scores. In comparison with the patients in the sham stimulation group, they showed an increase in right medial temporal metabolism between baseline and week 11. Furthermore, the week 2 images revealed that the metabolic change occurred early and was predictive of the clinical improvement, thus suggesting that PET scans can be used as a guide to individual therapeutic strategies and are capable of distinguishing responders and non-responders.

Taken together, these findings indicate that PET imaging may provide early neurobiological markers of treatment responsiveness. Region-specific metabolic changes detected during or shortly after the initiation of interventions such as rTMS could help distinguish responders from non-responders, thereby guiding the selection and personalization of non-pharmacological therapies, including cognitive-behavioral approach (Table 1, Table 2 and Table 3).

## 2. Discussion

FMS still represents a clinical enigma, but PET is reshaping the narrative. No longer just a diagnosis of exclusion, FMS is now revealing its complex neurobiological footprint through advanced imaging techniques that map the metabolic, neurotransmitter, and neuroinflammatory alterations underlying chronic pain and sensory dysfunction. A recent review has addressed neuroimaging in FMS more broadly [66].

Evidence from [18F]FDG PET studies indicates altered cerebral metabolism in key pain-processing regions, including the thalamus, insula, and limbic structures [31,32]. Notably, these metabolic differences are associated with treatment response, with poor responders showing significantly higher metabolic activity in areas such as the thalamus and lentiform nucleus [27]. These findings suggest that abnormal metabolic patterns in these regions may contribute to the chronicity and amplification of pain in FMS, highlighting potential targets for therapeutic interventions.

In parallel, studies utilizing dopaminergic tracers have revealed reduced dopamine receptor availability in regions integral to pain modulation, such as the ACC, fusiform gyrus, and striatum [53,54,55]. The observed inverse relationships between receptor BP and both spontaneous and evoked pain ratings support the hypothesis that dopaminergic dysfunction plays a crucial role in heightened pain sensitivity in FMS. This points to dopaminergic pathways not only as key contributors to the pathophysiology of FMS but also as potential biomarkers for predicting treatment outcomes [67,68].

Emerging research focused on neuroinflammation has further advanced our understanding of FMS. PET studies using TSPO-targeted tracers have consistently shown increased microglial activation in brain regions involved in both pain and emotional processing, such as the thalamus, ACC, and prefrontal cortex [43,44,45,46]. These neuroinflammatory markers have been correlated with clinical parameters such as pain intensity, stress levels, and even neurometabolic changes (e.g., creatine and glutamate levels) as well as peripheral biomarkers [69]. This integrated central–peripheral perspective underscores the role of neuroimmune dysregulation in central sensitization, a hallmark of FMS.

Accumulating evidence supports a unifying pathophysiological framework in which sustained peripheral immune activation—marked by elevated levels of cytokines, chemokines (e.g., IL-6, TNF-α), and gut-derived metabolites such as glutamate—modulates nociceptive processing via both humoral dissemination and neuroimmune reflex pathways [64,70,71,72]. These peripheral signals promote microglial and astrocytic activation within the central nervous system, driving maladaptive synaptic plasticity and persistent central sensitization [73,74,75]. Furthermore, disruption of blood–brain barrier integrity may facilitate the translocation of peripheral immune mediators into the central nervous system parenchyma, thereby exacerbating glial reactivity and sustaining neuroinflammation [76]. This neuroimmune crosstalk not only underlies the chronic and widespread pain phenotype of fibromyalgia but may also account for its phenotypic heterogeneity, suggesting the existence of pathobiological endotypes with distinct inflammatory signatures.

Moreover, post-treatment PET imaging studies, such as those assessing changes after high-frequency repetitive rTMS, have demonstrated that early metabolic changes in limbic regions may predict clinical improvements [65]. This finding suggests that PET imaging could serve not only as a diagnostic tool but also as a dynamic biomarker for monitoring therapeutic efficacy and guiding personalized treatment approaches.

In this context, PET imaging may help define biologically distinct subgroups of fibromyalgia patients based on patterns of neuroinflammation (e.g., TSPO expression), neurotransmitter dysregulation (e.g., serotonergic or dopaminergic transporter availability), or regional metabolic alterations. Such imaging-derived markers, when integrated with clinical and biochemical phenotyping, could support the identification of mechanistic endotypes and inform stratified treatment approaches.

The identification of pathophysiological subgroups within fibromyalgia may be operationalized through PET imaging by quantifying distinct neurobiological domains. A glial-driven inflammatory endotype may be characterized by elevated TSPO expression in regions implicated in pain modulation and affective regulation. Alternatively, subgroups defined by neuromodulatory dysfunction may exhibit altered binding potentials of serotonergic or dopaminergic transporters (e.g., DAT), reflecting impairments in monoaminergic signaling. In parallel, [18F]FDG PET—based assessments of cerebral glucose metabolism may delineate discrete metabolic phenotypes, including prefrontal hypometabolism and limbic hyperactivity, which correspond to cognitive-affective versus nociceptive symptom predominance. These imaging-derived profiles, when integrated with peripheral immune signatures and clinical phenotyping, offer a biologically grounded framework for the stratification of fibromyalgia into mechanistically distinct endotypes, with direct implications for treatment selection and trial enrichment.

Despite these promising findings, several challenges remain. While several PET studies have identified alterations in metabolic activity, neuroinflammation, and neurotransmitter systems in patients with FMS, the specificity of these patterns relative to other chronic pain conditions remains to be established. Moreover, the absence of standardized quantitative metrics limits the ability to calculate absolute changes in tracer uptake.

Interpretation of current PET evidence in fibromyalgia is limited by methodological heterogeneity and the absence of direct head-to-head comparisons with other chronic pain syndromes. While specific alterations—such as increased TSPO binding or reduced serotonergic transporter availability—have been consistently reported, these molecular changes are not unique to FMS and may reflect shared pathophysiological features across nociplastic, inflammatory, and neuropathic pain conditions. Future studies employing direct cross-condition comparisons (e.g., with chronic fatigue syndrome, irritable bowel syndrome, rheumatoid arthritis, and complex regional pain syndrome) are essential to isolate disease-specific neurobiological signatures.

Looking ahead, several advancements in PET technology could further enhance its role in FMS diagnosis and treatment monitoring. Improved radiotracers with higher specificity for pain-related neurobiological processes or more sensitive markers of neuroinflammation could lead to more accurate diagnosis and a better understanding of the mechanisms behind individual FMS presentations.

The heterogeneity of FMS, small sample sizes, and variability in imaging protocols limit the generalizability of current results. Differences in PET acquisition parameters, tracer selection, quantification methods, and region of interest definitions reduce reproducibility and hamper cross-study comparisons. The adoption of standardized imaging protocols is, therefore, essential to ensure methodological consistency, enhance data comparability, and support future multi-center research efforts. Additionally, the high cost and limited accessibility of advanced PET tracers pose significant barriers to widespread clinical implementation. Future research should focus on larger, more homogeneous cohorts and standardized imaging protocols to validate these biomarkers and facilitate their integration into routine clinical practice.

In terms of future management, PET imaging may become a pivotal tool in personalizing treatment for FMS patients. By identifying specific neurobiological alterations—whether metabolic dysregulation, dopaminergic dysfunction, or neuroinflammation—PET could guide clinicians in selecting the most appropriate therapies.

In addition to pharmacological interventions, PET imaging could inform the use of non-pharmacological treatments, such as TMS or cognitive-behavioral therapies, by identifying brain regions that may be most responsive to such therapies. Moreover, PET could be used to monitor the effectiveness of these treatments over time, providing real-time data on the brain’s response to various interventions and enabling clinicians to adjust treatment strategies as needed.

## 3. Conclusions

FMS is a highly heterogeneous and complex syndrome, marked by diverse clinical presentations and neuropathological alterations that hinder consistent characterization and differentiation from healthy individuals. PET imaging is emerging as a valuable tool to disentangle this complexity by revealing distinct neurobiological patterns that may support the identification of clinically meaningful subgroups. For example, some patients exhibit predominant neuroinflammatory activity, while others display dopaminergic dysregulation—suggesting divergent underlying mechanisms that may differentially influence treatment response.

## Figures and Tables

**Table 1:** Summary of available studies investigating PET/CT in fibromyalgia.

Author	Pathway	PET Radiotracer	Population	Main Findings
Usui	Glutamatergic system	[18F]FDG	18 FMS patients vs. 18 healthy controls	No significant group differences in global brain glucose metabolism. Patients with poor prognosis showed increased metabolism in the right thalamus, left lentiform nucleus, and right parahippocampal gyrus. Good prognosis was associated with hypometabolism in the left insula and lentiform nucleus. Parahippocampal activity correlated with Tender Point count.
Wood	Glutamatergic system	[18F]FDG	Single FMS patient	Reduced metabolic activity in the left insular cortex.
Walitt	Glutamatergic system	[18F]FDG	9 FMS patients (pre-/post-treatment)	Increased metabolism in limbic structures after an 8-week treatment, paralleling symptom improvement. No change detected in somatosensory regions.
Boyer	Glutamatergic system	[18F]FDG	38 FMS patients (rTMS intervention)	Increased metabolism in the right medial temporal lobe at week 11. Changes were positively correlated with clinical improvements in FIQ and SF-36 scores.
Wood	Dopaminergic system	[18F]DOPA	6 FMS patients vs. 8 controls	Reduced uptake in regions including the ventral tegmental area, substantia nigra, locus coeruleus, medial thalamus, hippocampus, ACC, and insular cortex.
Wood	Dopaminergic system	[11C]raclopride	11 FMS patients vs. 11 controls	During painful stimulation, dopamine release in the basal ganglia occurred in controls but not in FMS patients. FMS patients rated the pain as more intense.
Ledermann	Dopaminergic system	[11C]raclopride	11 FMS patients vs. 13 controls	Significantly reduced binding in the left ventral striatum, caudate nucleus, and nucleus accumbens in FMS patients.
Albrecht	Dopaminergic system	[18F]Fallypride	12 FMS patients vs. 11 controls	Lower binding potential in the ACC and fusiform gyrus. Pain sensitivity inversely correlated with binding in orbitofrontal and parahippocampal regions.
Pomares	GABAergic system	[11C]flumazenil	26 FMS patients vs. 25 controls	Increased GABA_A receptor availability in regions including the precuneus, superior frontal gyrus, angular gyrus, and occipital cortex. Receptor availability positively correlated with pain intensity and functional scores.
Seo	Neuroinflammation	[11C]PK11195	12 FMS patients vs. 11 controls	Higher TSPO binding in the pre/postcentral gyri, superior parietal lobule, and medial/superior frontal regions in FMS. Lower binding in the medulla, superior temporal gyrus, and amygdala. Positive correlations with stress and pain scores.
Harris	Opioid system	[11C]carfentanil	17 FMS patients vs. 17 controls	Reduced μ-opioid receptor availability in the bilateral nucleus accumbens, left amygdala, and right dorsal ACC. Binding inversely correlated with affective pain ratings.
Christina Mueller	Opioid system	[18F]DPA-714	15 FMS patients vs. 10 controls	Increased radioligand binding (V_T) in several brain regions, irrespective of TSPO binding status. Group differences in right parietal gray matter correlated with lower quality of life, higher pain, and cognitive symptoms.
Daniel S. Albrecht	Opioid system	[11C]PBR28	31 FMS patients vs. 27 controls	Elevated TSPO binding in FMS suggests glial activation. Absence of increased [11C]-L-deprenyl-D2 signal indicates microglial—rather than astrocytic—contribution to neuroinflammation. Larger studies are needed to clarify astrocytic involvement.

**Table 2:** PET Radiopharmaceuticals and Their Diagnostic Relevance in FMS.

Radiopharmaceutical	Clinical Relevance
[18F]FDG	Identifies regional metabolic changes; supports prognosis and treatment monitoring
[18F]DOPA	Reveals dopaminergic hypofunction; may identify patients with altered central pain modulation
[11C]raclopride	Detects impaired dopamine release during pain; may help identify patients with dysfunctional reward processing and guide tailored interventions
[18F]Fallypride	Shows reduced D2/D3 receptor binding; correlates with pain sensitivity and emotional dysregulation
[11C]flumazenil	Detects increased GABA_A receptor availability; associated with greater pain severity
[11C]PK11195	Indicates elevated TSPO binding; suggests neuroinflammation as a contributing mechanism
[18F]DPA-714	Marks glial activation; linked to pain intensity and cognitive impairment
[11C]PBR28	Reveals selective microglial activation; no astrocyte involvement, supporting a targeted neuroimmune response
[11C]carfentanil	Detects reduced μ-opioid receptor availability; reflects impaired endogenous pain inhibition

**Table 3:** Relationship Between PET Imaging Findings and Standardized Pain Measures in FMS Studies. Abbreviations: FMS = Fibromyalgia Syndrome; FIQ = Fibromyalgia Impact Questionnaire; SF-36 = Short Form-36 Health Survey; VAS = Visual Analogue Scale; QoL = Quality of Life; TSPO = Translocator Protein.

Radiopharmaceutical	Pain Measures Used	PET-Clinical Association
[18F]FDG	FIQ, SF-36, Tender Point Count	Parahippocampal metabolism correlated with tenderness; limbic metabolism changes paralleled symptom improvement.
[18F]DOPA	Subjective pain ratings	Reduced dopamine uptake in pain/reward regions associated with altered pain perception.
[11C]raclopride	VAS, Pain intensity scales	Reduced dopamine release during painful stimulation linked to higher pain ratings.
[18F]Fallypride	Pain sensitivity scores	Binding inversely correlated with pain sensitivity in orbitofrontal and parahippocampal regions.
[11C]flumazenil	FIQ, Pain intensity	Increased receptor availability positively correlated with pain and dysfunction.
[11C]PK11195	VAS, Perceived Stress Scale	TSPO binding levels correlated with pain and stress scores.
[18F]DPA-714	Pain, QoL, cognitive symptoms	Higher radioligand binding associated with greater pain and lower quality of life.
[11C]PBR28	VAS, stress measures	TSPO elevation consistent with microglial activation and pain/stress correlation.
[11C]carfentanil	Affective pain ratings	Reduced μ-opioid receptor availability inversely correlated with affective pain.

## Abbreviations

The following abbreviations are used in this manuscript:

[18F]FDG	[18F]fluorodeoxyglucose
ACC	anterior cingulate cortex
BP	binding potential
DATs	Presynaptic DA transporters
FMS	Fibromyalgia Syndrome
MDD	major depressive disorder
NMD	N-methyl-D-aspartate
PET	Positron Emission Tomograph
rTMS	transcranial magnetic stimulation
SPM	Statistical parametric mapping
VTA	ventral tegmental area
